# The MR neuroimaging protocol for the Accelerating Medicines Partnership® Schizophrenia Program

**DOI:** 10.1038/s41537-025-00581-6

**Published:** 2025-04-02

**Authors:** Michael P. Harms, Kang-Ik K. Cho, Alan Anticevic, Nicolas R. Bolo, Sylvain Bouix, Dylan Campbell, Tyrone D. Cannon, Guillermo Cecchi, Mathias Goncalves, Anastasia Haidar, Dylan E. Hughes, Igor Izyurov, Omar John, Tina Kapur, Nicholas Kim, Elana Kotler, Marek Kubicki, Joshua M. Kuperman, Kristen Laulette, Ulrich Lindberg, Christopher Markiewicz, Lipeng Ning, Russell A. Poldrack, Yogesh Rathi, Paul A. Romo, Zailyn Tamayo, Cassandra Wannan, Alana Wickham, Walid Yassin, Juan Helen Zhou, Jean Addington, Luis Alameda, Celso Arango, Nicholas J. K. Breitborde, Matthew R. Broome, Kristin S. Cadenhead, Monica E. Calkins, Eric Yu Hai Chen, Jimmy Choi, Philippe Conus, Cheryl M. Corcoran, Barbara A. Cornblatt, Covadonga M. Diaz-Caneja, Lauren M. Ellman, Paolo Fusar-Poli, Pablo A. Gaspar, Carla Gerber, Louise Birkedal Glenthøj, Leslie E. Horton, Christy Lai Ming Hui, Joseph Kambeitz, Lana Kambeitz-Ilankovic, Matcheri S. Keshavan, Sung-Wan Kim, Nikolaos Koutsouleris, Jun Soo Kwon, Kerstin Langbein, Daniel Mamah, Daniel H. Mathalon, Vijay A. Mittal, Merete Nordentoft, Godfrey D. Pearlson, Jesus Perez, Diana O. Perkins, Albert R. Powers, Jack Rogers, Fred W. Sabb, Jason Schiffman, Jai L. Shah, Steven M. Silverstein, Stefan Smesny, William S. Stone, Gregory P. Strauss, Judy L. Thompson, Rachel Upthegrove, Swapna K. Verma, Jijun Wang, Daniel H. Wolf, Rene S. Kahn, John M. Kane, Patrick D. McGorry, Barnaby Nelson, Scott W. Woods, Martha E. Shenton, Stephen J. Wood, Carrie E. Bearden, Accelerating Medicines Partnership® Schizophrenia (AMP® SCZ), Michael P. Harms, Kang-Ik K. Cho, Alan Anticevic, Nicolas R. Bolo, Sylvain Bouix, Dylan Campbell, Tyrone D. Cannon, Guillermo Cecchi, Mathias Goncalves, Anastasia Haidar, Dylan E. Hughes, Igor Izyurov, Omar John, Tina Kapur, Nicholas Kim, Elana Kotler, Marek Kubicki, Joshua M. Kuperman, Kristen Laulette, Ulrich Lindberg, Christopher Markiewicz, Lipeng Ning, Russell A. Poldrack, Yogesh Rathi, Paul A. Romo, Zailyn Tamayo, Cassandra Wannan, Alana Wickham, Walid Yassin, Juan Helen Zhou, Jean Addington, Luis Alameda, Celso Arango, Nicholas J. K. Breitborde, Matthew R. Broome, Kristin S. Cadenhead, Monica E. Calkins, Eric Yu Hai Chen, Jimmy Choi, Philippe Conus, Cheryl M. Corcoran, Barbara A. Cornblatt, Covadonga M. Diaz-Caneja, Lauren M. Ellman, Paolo Fusar-Poli, Pablo A. Gaspar, Carla Gerber, Louise Birkedal Glenthøj, Leslie E. Horton, Christy Lai Ming Hui, Joseph Kambeitz, Lana Kambeitz-Ilankovic, Matcheri S. Keshavan, Sung-Wan Kim, Nikolaos Koutsouleris, Jun Soo Kwon, Kerstin Langbein, Daniel Mamah, Daniel H. Mathalon, Vijay A. Mittal, Merete Nordentoft, Godfrey D. Pearlson, Jesus Perez, Diana O. Perkins, Albert R. Powers, Jack Rogers, Fred W. Sabb, Jason Schiffman, Jai L. Shah, Steven M. Silverstein, Stefan Smesny, William S. Stone, Gregory P. Strauss, Judy L. Thompson, Rachel Upthegrove, Swapna K. Verma, Jijun Wang, Daniel H. Wolf, Rene S. Kahn, John M. Kane, Patrick D. McGorry, Barnaby Nelson, Scott W. Woods, Martha E. Shenton, Stephen J. Wood, Carrie E. Bearden, Ofer Pasternak, Ofer Pasternak

**Affiliations:** 1 Department of Psychiatry, Washington University School of Medicine, St. Louis, MO USA; 2 Department of Psychiatry, Brigham and Women’s Hospital, Harvard Medical School, Boston, MA USA; 3 Department of Psychiatry, Yale University School of Medicine, New Haven, CT USA; 4 Department of Psychiatry, Beth Israel Deaconess Medical Center, Harvard Medical School, Boston, MA USA; 5 Department of Software Engineering and Information Technology, École de technologie supérieure, Montréal, QC Canada; 6 Department of Psychology, Yale University, New Haven, CT USA; 7 T.J. Watson Research Laboratory, IBM Research, Yorktown Heights, NY USA; 8 Department of Psychology, Stanford University, Stanford, CA USA; 9 Department of Psychology, University of California, Los Angeles, CA USA; 10 Department of Psychiatry and Psychotherapy, Jena University Hospital, Jena, Germany; 11 Massachusetts General Hospital, Harvard Medical School, Boston, MA USA; 12 Department of Radiology, Brigham and Women’s Hospital, Harvard Medical School, Boston, MA USA; 13 Department of Radiology, University of California, San Diego, CA USA; 14 Department of Clinical Physiology and Nuclear Medicine, Copenhagen University Hospital - Rigshospitalet, Glostrup, Denmark; 15 Seaman Family MR Research Centre, Calgary, AB Canada; 16 Orygen, Parkville, Victoria Australia; 17 Centre for Sleep and Cognition and Centre for Translational MR Research, Yong Loo Lin School of Medicine, National University of Singapore, Singapore, Singapore; 18 Department of Psychiatry, Hotchkiss Brain Institute, University of Calgary, Calgary, AB Canada; 19 General Psychiatry Service, Treatment and Early Intervention in Psychosis Program, Lausanne University Hospital and University of Lausanne, Lausanne, Switzerland; 20 Department of Child and Adolescent Psychiatry, Institute of Psychiatry and Mental Health, Hospital General Universitario Gregorio Marañón, Instituto de Salud Carlos III, School of Medicine, Universidad Complutense, Madrid, Spain; 21 Department of Psychiatry and Behavioral Health, Ohio State University Wexner Medical Center, Columbus, OH USA; 22 Department of Psychology, Ohio State University, Columbus, Ohio USA; 23 Institute for Mental Health, School of Psychology, University of Birmingham, Birmingham, UK; 24 Birmingham Womens and Childrens NHS Foundation Trust, Birmingham, UK; 25 Department of Psychiatry, University of California, San Diego, CA USA; 26 Department of Psychiatry, Perelman School of Medicine, University of Pennsylvania, Philadelphia, PA USA; 27 Department of Psychiatry, School of Clinical Medicine, Li Ka Shing Faculty of Medicine, University of Hong Kong, Hong Kong, China; 28 Institute of Mental Health, Singapore, Singapore; 29 Olin Neuropsychiatry Research Center, Hartford HealthCare Behavioral Health Network, Hartford, CT USA; 30 Department of Psychiatry, Icahn School of Medicine at Mount Sinai, New York, NY USA; 31 Department of Psychiatry, Donald and Barbara Zucker School of Medicine at Hofstra/Northwell, Hempstead, NY USA; 32 Institute of Behavioral Science, Feinstein Institute for Medical Research, Manhasset, NY USA; 33 Department of Psychology & Neuroscience, Temple University, Philadelphia, PA USA; 34 Department of Psychosis Studies, King’s College London, London, UK; 35 Department of Brain and Behavioral Sciences, University of Pavia, Pavia, Italy; 36 Department of Psychiatry, University of Chile, Santiago, Chile; 37 Prevention Science Institute, University of Oregon, Eugene, OR USA; 38 Oregon Research Institute, Springfield, OR USA; 39 Copenhagen Research Centre for Mental Health, University of Copenhagen, Copenhagen, Denmark; 40 Department of Psychiatry, University of Pittsburgh School of Medicine, Pittsburgh, PA USA; 41 Department of Psychiatry, Faculty of Medicine and University Hospital Cologne, University of Cologne, Cologne, Germany; 42 Department of Psychiatry, Chonnam National University Medical School, Gwangju, Korea; 43 Department of Psychiatry and Psychotherapy, Ludwig-Maximilian-University Munich, Munich, Germany; 44 Department of Psychiatry, Seoul National University College of Medicine, Seoul, Korea; 45 Department of Psychiatry and Behavioral Sciences, Weill Institute for Neurosciences, University of California, San Francisco, CA USA; 46 Mental Health Service, Veterans Affairs San Francisco Health Care System, San Francisco, CA USA; 47 Department of Psychology, Northwestern University, Evanston, IL USA; 48 Department of Clinical Medicine, Copenhagen University Hospital, Copenhagen, Denmark; 49 Early Intervention in Psychosis Service, Cambridgeshire and Peterborough NHS Foundation Trust, Cambridge, UK; 50 Institute of Biomedical Research, Department of Medicine, Universidad de Salamanca, Salamanca, Spain; 51 Department of Psychiatry, University of North Carolina at Chapel Hill, Chapel Hill, NC USA; 52 Connecticut Mental Health Center, New Haven, CT USA; 53 Centre for Human Brain Health, University of Birmingham, Birmingham, UK; 54 Department of Psychological Science, University of California, Irvine, CA USA; 55 Douglas Research Centre, McGill University, Montreal, Canada; 56 Department of Psychiatry, McGill University, Montreal, Canada; 57 Department of Psychiatry, University of Rochester Medical Center, Rochester, NY USA; 58 Department of Psychology, University of Georgia, Athens, CA USA; 59 Department of Neuroscience, University of Rochester Medical Center, Rochester, NY USA; 60 Duke-National University of Singapore Medical School, Singapore, Singapore; 61 Shanghai Mental Health Center, Shanghai Jiaotong University School of Medicine, Shanghai, China; 62 Centre for Youth Mental Health, The University of Melbourne, Parkville, VIC Australia; 63 Department of Psychiatry and Biobehavioral Sciences, Semel Institute for Neuroscience and Human Behavior, University of California, Los Angeles, CA USA

**Keywords:** Biomarkers, Psychosis

## Abstract

Neuroimaging with MRI has been a frequent component of studies of individuals at clinical high risk (CHR) for developing psychosis, with goals of understanding potential brain regions and systems impacted in the CHR state and identifying prognostic or predictive biomarkers that can enhance our ability to forecast clinical outcomes. To date, most studies involving MRI in CHR are likely not sufficiently powered to generate robust and generalizable neuroimaging results. Here, we describe the prospective, advanced, and modern neuroimaging protocol that was implemented in a complex multi-site, multi-vendor environment, as part of the large-scale Accelerating Medicines Partnership® Schizophrenia Program (AMP® SCZ), including the rationale for various choices. This protocol includes T1- and T2-weighted structural scans, resting-state fMRI, and diffusion-weighted imaging collected at two time points, approximately 2 months apart. We also present preliminary variance component analyses of several measures, such as signal- and contrast-to-noise ratio (SNR/CNR) and spatial smoothness, to provide quantitative data on the relative percentages of participant, site, and platform (i.e., scanner model) variance. Site-related variance is generally small (typically <10%). For the SNR/CNR measures from the structural and fMRI scans, participant variance is the largest component (as desired; 40–76%). However, for SNR/CNR in the diffusion scans, there is substantial platform-related variance (>55%) due to differences in the diffusion imaging hardware capabilities of the different scanners. Also, spatial smoothness generally has a large platform-related variance due to inherent, difficult to control, differences between vendors in their acquisitions and reconstructions. These results illustrate some of the factors that will need to be considered in analyses of the AMP SCZ neuroimaging data, which will be the largest CHR cohort to date.

Watch Dr. Harms discuss this article at https://vimeo.com/1059777228?share=copy#t=0.

## Introduction

Magnetic resonance imaging (MRI) studies of patients with schizophrenia consistently indicate, on average, reduced gray matter volume and cortical thinning across multiple brain regions^1,2^, white matter microstructural alterations^3–6^ and disruptions in neural activation and connectivity between distributed brain regions^7–9^. Notably, subtle changes in brain structure and connectivity are present at the onset of the first episode of psychosis, and evidence indicates that they may even precede the onset of frank psychosis^10–17^.

The first psychotic episode of schizophrenia is typically preceded by a prodromal period involving nonpsychotic as well as subthreshold psychotic-like symptomatology and a decline in social and role functioning^18–20^. Efforts to proactively and reliably identify individuals who may eventually develop psychosis led to the development of clinical research criteria for the clinical high risk for psychosis (CHR) syndrome^21–23^. Recent meta-analysis indicates that about 15% of individuals meeting CHR criteria progress to frank psychosis by 1 year, 20% by 2 years, 30% by 4 years, and 35% by 10 years^24^. Remission from CHR status occurs in ~40% at 2 years, while others experience persistent attenuated psychotic symptoms and/or develop other mental disorders^25,26^. As such, there has been particular emphasis on identifying specific prognostic or predictive biomarkers that can enhance our ability to forecast clinical outcomes, elucidate biological processes associated with the trajectories to those outcomes (for a comprehensive review, see ref.^27^), and ultimately to alleviate the high burden associated with psychotic disorders^28^. To further these aims, the Accelerating Medicines Partnership® Schizophrenia Program (AMP® SCZ), launched in 2020, is the largest public-private partnership that intends to develop biomarker algorithms to predict individual clinical trajectory and outcomes, with the overarching aim to improve our understanding of psychosis-relevant disorder pathways and to identify new and better targets for treatment. AMP SCZ includes two research networks, the ProNET and PRESCIENT consortia, which collect data from CHR individuals and community controls, and the Data Processing, Analysis and Coordination Center (DPACC). AMP SCZ will be the largest coordinated study of CHR to date, with a target of ~2000 CHR individuals and over 600 community controls^26,29^.

Adding MRI markers into clinically-based prediction algorithms may improve prediction of individual outcomes^30–32^. One promising imaging biomarker that may improve prediction in CHR is accelerated thinning of cortical gray matter, detectable via T1-weighted (T1w) structural MRI. Some studies have observed a more rapid decline in cortical thickness in CHR individuals who eventually transition to psychosis compared to those who do not, most consistently in frontal and temporal brain regions^33–38^. Additional findings indicate that the accelerated cortical thinning that occurs prior to onset of frank psychosis can be detected within a brief follow-up period of (on average) 3 months^39^. Notably, adding this cortical thinning metric significantly improved the performance of an individual risk calculator for prediction of conversion to psychosis, particularly for individuals who had experienced a shorter duration (<120 days) of subthreshold psychotic symptoms [area under the curve (AUC) increased from 0.71 to 0.84]^40^. Moreover, this decrease in thickness was not linked to the use of antipsychotic medications. Additional measures derived from anatomical T1w or T2-weighted (T2w) MRI, such as gyrification^41–43^, T1w divided by T2w (“myelin”) ratio maps^44–47^, and structural covariance networks^48–50^ are also promising, as these measures can reveal various informative morphological changes associated with the pathophysiology of psychosis and schizophrenia.

Likewise, as schizophrenia has long been conceptualized as a disorder of distributed brain dysconnectivity^51–53^, investigation of intrinsic low frequency blood-oxygen-level-dependent (BOLD) signal fluctuations in the brain, via resting state functional MRI (rfMRI), has emerged as a valuable non-invasive approach for probing the function of large-scale neural networks. Disrupted functional interactions between the cortex and the thalamus in particular have been consistently observed in individuals with schizophrenia^54–58^ and proposed to underlie the array of characteristic cognitive and clinical symptoms^59^. Similar to patterns observed in individuals with established illness^60^, widespread hypoconnectivity between the thalamus and prefrontal and cerebellar regions, concomitant with thalamic hyperconnectivity with sensorimotor cortex, was observed in CHR individuals at baseline, most prominently among those who subsequently converted to psychosis^61^. In a comparison of CHR to healthy control individuals, Colibazzi et al.^62^ also found hyperconnectivity of thalamus, albeit to a set of perisylvian clusters that included the auditory cortex. More recently, Collin et al.^63^ found that CHR individuals who went on to develop psychosis had broadly abnormal network organization at baseline, implicating more than just the thalamus in altered brain functional connectivity patterns. These findings suggest that functional changes in brain network organization precede the onset of psychosis and may influence the development of psychosis in those at CHR.

Fewer studies have investigated white matter or structural connectivity abnormalities, assessed via diffusion-weighted MRI (dMRI), as potential markers of conversion in CHR individuals, and findings of existing studies are mixed. Similar to rfMRI findings, alterations in thalamo-cortical white matter connectivity have been noted both in individuals with schizophrenia^64^, sub-threshold psychosis^65^, and in individuals at CHR^66^. While some studies have observed alterations in dMRI derived measures such as lower fractional anisotropy (FA) and higher extracellular free-water in CHR converters compared to those who do not convert to psychosis^67–69^, others suggest that white matter abnormalities characterize the CHR state regardless of a later conversion to psychosis^16,70^.

Thus, there are many open questions regarding meso- and macro-scale brain changes in the CHR syndrome, including the timing of any changes in these imaging phenotypes, their sensitivity and specificity, and the robustness of their ability to predict outcomes (e.g., conversion, remission), both independently, across MRI modalities, and in combination with other measures of interest. For these reasons the AMP SCZ initiative included neuroimaging at the baseline and 2 month follow-up timepoints. The imaging protocol collects T1w, T2w, rfMRI, and dMRI scans using an advanced, modern protocol motivated by the principles of the Human Connectome Project^71^ for the first time in a large-scale CHR study. Here we provide an overview of the AMP SCZ neuroimaging protocol, including a description of how the study protocol was developed, the rationale for various choices, and preliminary cross-site, cross-vendor comparisons of various quantitative quality control measures, to assess consistency across sites/vendors at the stage of initial acquisition.

## Background

### Protocol development

Development of the MR imaging protocol for AMP SCZ began in early November 2020 with the formation of a working group with expertize in multiple MR modalities and with representation from both the ProNET and PRESCIENT consortia, the DPACC and other experts from AMP SCZ partners. The workgroup aimed to develop an imaging protocol that included scans that could contribute to the analytic goals of AMP SCZ and that was applicable for the CHR population, while incorporating recent advances such as simultaneous multi-slice imaging^72,73^ in a multi-vendor study. Thus, potential challenges in implementing the protocol in a future clinical framework or the generalizability of the protocol beyond a research environment were not considered. A primary challenge was that across the two consortia there was considerable variety in the imaging platforms that needed to be accommodated (Table 1). Therefore, the workgroup aimed to implement MRI protocols with a reasonable degree of harmonization on Siemens, General Electric (GE), and Philips imaging platforms.

**Table 1:** Scanning platforms.

Platform	Receive coil	Imaging sites (ProNET, PRESCIENT)	Software versions (at site initiation)	Max gradient strength (per axis; mT/m)
Siemens Prisma	32 ch Head or 64 ch HeadNeck^a^	30 (23, 7)	VE11{B,C,E}, XA30	80
Siemens Skyra	32 ch Head	3 (2, 1)	VE11{C,E}	45
Siemens Vida	64 ch HeadNeck	1 (0, 1)	XA31	60
GE MR750	Nova 32 ch or 32 ch Head (GE)^b^	3 (3, 0)	DV26.0_R{03,05,06}	50
Philips Achieva dStream DDAS	Philips 32 ch	1 (0, 1)	5.7.1	Standard mode: 40 Enhanced mode: 62^c^

^a^Siemens sites with both the 64ch and 32ch coils were instructed to use the 32ch coil. Seven Siemens sites are using the 64 ch coil.

^b^The Nova 32 ch coil was preferred for the GE sites, but one site only had the standard GE 32 ch head coil at the start of the study.

^c^The “Quasar Dual” gradient system on the Achieva can be operated in two different modes, which trade maximum gradient strength for slew rate. The dMRI scans were collected with ‘enhanced’ mode (62 mT/m gradients at a reduced 100 T/m/s slew rate); all other scans were collected with the standard gradient mode (40 mT/m gradients at a 200 T/m/s slew rate).

As a starting point for creating the AMP SCZ neuroimaging protocol, we reviewed the protocols developed for the Human Connectome Project (HCP)^74^ and the Adolescent Brain Cognitive Development (ABCD) Study^75^—two other large, multi-site studies whose imaging protocols have served as templates for many other studies^76,77^. The HCP-Lifespan protocol was designed for a Siemens Prisma platform, and the ABCD Study has protocols for Siemens, GE and Philips platforms. We decided to target a protocol with 45–50 min of scanning, so that the protocol could be completed in a 1 h scheduled slot, which is well-tolerated by the clinical population included in the study. We also decided that the imaging modalities targeted would be T1w and T2w structural scans, functional MRI, and multi-shell diffusion MRI, so that the study would have the flexibility to generate measures of structural morphometry (e.g., volume, thickness, and area), connectivity (both functional and structural), and white matter microstructure – the same measures that have been the predominant focus of previous CHR studies and which show considerable promise in the literature (see Introduction).

After surveying the sites, we learned that most already had the necessary licenses and software for simultaneous multi-slice (SMS, i.e., “multi-band”) imaging, so we decided early to use SMS for the rfMRI and dMRI scans, similar to the HCP and ABCD protocols. Further, most of the Siemens sites already had installed the multi-band (MB) sequence package available from the Center for Magnetic Resonance Research (CMRR) via a “Core Competence Partnership” (C2P) agreement with the University of Minnesota (UMN), so we opted to use the MB sequences and accompanying custom reconstruction code from CMRR for the fMRI and dMRI scans on all Siemens platforms because: (1) the CMRR MB implementation has a strong user base and a history going back over 10 years; (2) it provides valuable additional features, including options to output the “single-band reference image” and to rigorously change the phase-encoding polarity via a “Sequence:Special” flag (The approach for switching phase-encoding polarity in Siemens product EPI sequences suffers from a long-standing bug in which the phase-encoding and frequency axes themselves get swapped (e.g., instead of switching from “AP” to “PA” polarity, you can end up with a “RL” phase-encoding, if the operators are not careful); (3) in our estimation, the use of the CMRR MB sequences gave us the best likelihood of achieving consistent acquisitions and reconstructions across different Siemens platforms (including the VE11/XA30 software transition), given that the CMRR development team is sensitive to the importance of continuity and backwards compatibility as they adapt to different Siemens software versions. Siemens sites that did not already have the necessary C2P agreement with UMN were assisted with that process. Similarly, multi-band functionality was not native to the DV26 software level of the GE MR750 scanners, so the GE sites were assisted with obtaining the applications package (i.e., “Advanced Technology Software Module”) for the ABCD Study, which provided the multi-band functionality on the GE platform.

#### T1w and T2w structural scans

We opted for the 0.8 mm isotropic resolution used in the HCP protocol (rather than the 1.0 mm of the ABCD Study), since modern 32 channel (or greater) receive-coils are able to generate good quality data at the higher spatial resolution. However, given the number of Siemens sites involved, many of which did not already have a C2P agreement with Massachusetts General Hospital for use of their real-time motion corrected structural sequences with volumetric navigators (used by both HCP-Lifespan and ABCD Study protocols), we decided to use Siemens product sequences (i.e., no navigators) for acquisition of the structural scans. Concurrently, we disabled the analogous real-time motion correction feature (“PROMO”) in the structural scans within the ABCD applications package that the GE sites obtained. Similarly, we used product Philips T1w and T2w structural sequences without any real-time motion correction.

#### fMRI

Since fMRI is not particularly dependent on peak gradient strength, we opted for at least superficial harmonization of the basic fMRI acquisition parameters across all vendors and platforms (e.g., same TR/TE). We included 20 min of “resting-state” fMRI (rfMRI), as four 5-min runs, with two runs having “AP” phase-encoding polarity, and the other two having “PA” polarity, so that the rfMRI data would not be biased toward a particular polarity. We chose to focus our available time for fMRI solely on collecting rfMRI scans given the challenges in relating task fMRI measures to individual differences in a reliable manner for typical task fMRI scan durations^78–81^. We considered using passive viewing of movie clips or other visual stimuli (e.g., “Inscapes”^82^) for the fMRI scans as a more engaging condition for the participant. Also, compared to resting-state, connectomes obtained during movie watching may promote better prediction of individual differences in behavioral measures (e.g., cognition and emotion)^83^ and higher intra-subject connectome-based identifiability^84^. However, due to variability in video projection capabilities across sites, as well as implementational simplicity, we decided to stick with traditional viewing of a fixation cross for the rfMRI scans. Similarly, to manage the complexity of the protocol implementation and data acquisition across sites and vendors, we decided to not include collection of either physiological data (pulse oximetry and respiratory traces, used in certain denoising approaches) or eye tracking/recording (for potential quantification of arousal/sleepiness) as part of the fMRI acquisitions.

#### dMRI

Unlike fMRI, dMRI is highly dependent on peak gradient strength^85^, so implementing a strictly harmonized set of basic dMRI parameters across scanners would have appreciably limited the achievable performance of the Siemens Prisma scanners, which represent 79% of the sites (Table 1). Also, inspecting data from previous studies such as ABCD, we observed that scanners that do not have peak gradient strength similar to a Prisma scanner would not produce high quality data for high b-values. Given that, we adopted a “tiered” dMRI protocol, with “Tier 1” for the Prisma scanner designed to take full advantage of the Prisma’s capabilities (and potentially of other scanners with similar gradient capability), and a “Tier 2” dMRI protocol that is designed to work for all the other platforms by modifying key scanning parameters. Both tiers include a multi-shell dMRI acquisition with interleaved shells of *b* = 200, 500, 1000, and 2000 s/mm^2^ (*n* = 6, 10, 50, 50 volumes, respectively), but the Tier 1 protocol additionally includes an interleaved *b* = 3000 s/mm^2^ shell (*n* = 50 volumes). Notably, the diffusion-sensitizing directions for the *b* ≤ 2000 s/mm^2^ shells are the same for both tiers, so that it will be possible to analyze a subset of the Tier 1 dMRI data matched to the Tier 2 dMRI scans on diffusion directions and b-value if so desired (albeit still acquired with an appreciably shorter TE on the Tier 1). Directions in the shells were distributed over whole spheres. The specific diffusion-sensitizing directions for the *b* = 3000 and 2000 s/mm^2^ shells of the Tier 1 protocol were taken from the higher and lower (respectively) shells of the two shell HCP-Lifespan protocol, thus facilitating potential comparison with HCP-Lifespan data. The *b* = 1000 s/mm^2^ shell used the same directions as the *b* = 3000 s/mm^2^ shell, since estimation of the radial aspect of the diffusion propagator benefits from having the same directions collected at more than one b-value^86,87^. The *b* = 500 and 200 s/mm^2^ shells came from dodecahedron and icosahedron sampling schemes, respectively.

Table 2 summarizes the protocol and lists the default scan order. Detailed parameters for each modality are available in a Supplemental spreadsheet.

**Table 2:** Imaging protocol for AMP SCZ.

Scan(s)	Phase-encoding polarity	Resolution (mm)^a^	Volumes	Duration (min:s)^b^	Miscellaneous
Localizers					
Distortion Maps^c^	AP & PA	2.4	1–2 ^d^	2 × 0:06-0:14	
T1w structural	AP	0.8		6:19–6:54	2x phase acceleration
T2w structural	AP	0.8		5:22-6:21	2x phase acceleration
Distortion Maps	AP & PA	2.4	1–2	2 × 0:06-0:14	
Resting-state fMRI	AP & PA	2.4	2 × 333-343	2 × 5:04-5:12	TR/TE = 900/35 ms; Flip = 52^o^; MB = 6
dMRI, *b* = 0^e^	AP	1.8	7 or 9	0:37-0:56	
dMRI, Tier 1 or dMRI, Tier 2	PA	1.8	177 or 127-128	9:41 or 8:42-9:17	*b* = 0 (*n* = 11), 200 (6), 500 (10), 1000 (50), 2000 (50) s/mm^2^ in Tier 2; Tier 1 (Prisma) additionally includes *b* = 3000 (50); MB = 3
dMRI, *b* = 0	AP	1.8	7 or 9	0:37-0:56	
Distortion Maps	AP & PA	2.4	1–2	2 × 0:06-0:14	
Resting-state fMRI	AP & PA	2.4	2 × 333-343	2 × 5:04-5:12	
	Cumulative scan duration			46–48 min	

^a^Isotropic spatial resolution (voxel size).

^b^Durations reflect what is listed on the scanner, which includes calibration and discarded scans.

^c^A pair of spin-echo EPI scans with acquisition parameters (other than TR/TE) matched as closely as possible to the rfMRI scans. These scans are used for correction of the susceptibility-induced distortions in the gradient-echo rfMRI scans.

^d^Ranges reflect minor differences in the protocol across the different vendors. pair of spin-echo EPI scans with acquisition parameters (other than TR/TE) matched as closely as possible to the rfMRI scans. These scans are used for correction of the susceptibility-induced distortions in the gradient-echo rfMRI scans.

^e^The “*b* = 0” scans have identical parameters to the main dMRI scan, except they are acquired with opposite phase-encoding polarity, for use in the correction of the susceptibility distortions in the dMRI data^138^. The final volume of the “*b* = 0” scans was actually collected with the same maximum *b*-value as the main dMRI scan (2000 or 3000 s/mm^2^) to ensure that the internal sequence timing would be the same.

*AP* anterior to posterior phase-encoding direction; *PA* posterior to anterior, *MB* multi-band factor.

We considered the potential benefits of scanning “traveling human volunteers” at participating sites, which would include (1) facilitating site training and consistent scanning procedures across sites, (2) quantifying reliability or generalizability as well as the impact of site- and vendor-related variance^88–91^, and (3) their potential use in post-acquisition harmonization approaches^92–94^. However, given the number of sites, their geographic dispersion, and travel restrictions during the COVID-19 pandemic, we concluded that a traveling volunteer program was not feasible. Instead, and following the recommendation of the neuroimaging group, each site is locally recruiting demographically well-matched community control participants, approximately a third of whom will receive a repeat biomarker assessment.

### Site qualification and training

To qualify for participation in the study, sites first had to run the protocol on a phantom, which was then reviewed for expected acquisition parameters (see *Intake and automated QC review)*. Sites then scanned a human volunteer through the full MR protocol, which was again reviewed for protocol fidelity and additionally image quality. As part of this process, sites were required to send the volunteer session to their respective network (ProNET/PRESCIENT) hubs rather than to the DPACC directly, to confirm that the entire transfer chain of the MR data from site to hub to DPACC was operational prior to scanning participants.

Scan sessions are conducted with two individuals present at all times – one designated as the scan ‘Operator’ and the other as the ‘Assistant’. At some sites (13 sites for ProNET, 9 for PRESCIENT), the ‘Operator’ is a professionally trained individual (e.g., MR Technician, radiographer or radiologist), but that was not a requirement. At the initiation of the study, the neuroimaging team leadership hosted two Zoom sessions to review expectations and the details of running the MR protocol with the participating sites. Staff that join a site after initial site qualification are expected to read the standard operating procedures (SOPs), watch the recorded Zoom session from the initiation of the study, and participate in at least two scan sessions under the direct supervision of experienced staff prior to fulfilling either the Operator or Assistant role independently.

### Intake and automated Quality Control (QC) review

The MRI data for AMP SCZ goes through a series of QC steps, starting at the scanner itself, where the Operator and Assistant are instructed to view each collected dataset in real-time, provide a quality score for each scan, and repeat scans when severe image problems are identified. The DPACC developed a quality control pipeline, called Quick Quality Control (QQC) (https://github.com/AMP-SCZ/qqc) that is automatically applied on all incoming data and detects protocol deviations in the newly acquired AMP SCZ MRI data. The MRI data acquired from each site are transferred to their respective network hubs, then to a server controlled by the NIMH Data Archive, where they are accessible to the DPACC through the real-time dataflow system^29,95^. Upon arrival, QQC arranges the DICOM files and executes Heudiconv (v. 0.11.3) (10.5281/zenodo.6544633) and ‘dcm2niix’ (v1.0.20230121)^96^ to automatically convert the DICOMs into NIFTI files according to AMP SCZ specific settings and with preset file names in a BIDS organizational structure^97^. During this conversion process, scan parameters and other information in the DICOMs are extracted and stored in sidecar JSON files. These parameters are then automatically compared with templates of approved parameters for each site to assure consistency and proper data acquisition.

Other tests in the QQC include comparison of the number of diffusion weighting directions and diffusion weighting values. The number of volumes and slices, and their orientation, are compared in all acquisition series, which detects conversion issues, partial data transfer, incomplete scans, and scans with inconsistent slice orientations. On the Siemens platforms, QQC also confirms that the shim was consistent across scans (via the ‘ShimSetting’ field returned by ‘dcm2niix’), which is a very valuable check for identifying sessions that were not acquired according to the instructions in the MRI Acquisition SOP.

QQC generates an HTML report for all tested variables and alerts the DPACC MRI team via email when deviations exist. The report also includes multi-slice (mosaic-style) snapshots of the imaging data for all modalities, and figures of the mean intensity across volumes for the fMRI and dMRI data, which provide simple but useful visual information for detecting major image artifacts (e.g., large distortions). The whole process from the arrival to the DPACC servers of new data to the generation of QQC summary email takes less than 20 min for a full AMP SCZ MRI session. Thus, QQC enables immediate highlighting of any protocol deviations and allows the DPACC to promptly address and prevent recurrence of problems in subsequent sessions at the site.

The QQC report also includes a copy of the “MRI Run Sheet”. This is an online form that captures relevant information from the scan session (in REDCap for ProNET, and RPMS for PRESCIENT). This form is ideally completed by the MR Assistant in real-time during scanning, and prompts the Assistant to include annotations of any problems or variances (e.g., reasons for omission or re-run of any specific scan), the Operator’s immediate assessment of the scan quality, and the alertness of the participant during the rfMRI scans. The details of this Run Sheet are customized to each scanner vendor to reflect the specifics of the protocol for that vendor, including notes to remind the scan team in real-time of salient specifics for the execution of the MR protocol on that platform. The Run Sheet is reviewed as part of the intake of each session. In cases of problems identified via QQC that are not explained via the Run Sheet, sites are contacted to better understand what occurred, remind the sites regarding protocol expectations, and have sites retroactively augment their Run Sheet entries to document problems appropriately. Additionally, all issues are logged in a network specific tracker, that ensures items requiring follow-up are captured and addressed.

Finally, QQC also initiates the next processing steps by executing MRIQC^98^, FreeSurfer^99,100^, fMRIPrep^101^, and an in-house diffusion preprocessing pipeline (modeled after the HCPpipelines). From these pipelines, measures like signal- and contrast-to-noise ratios, spatial smoothness and head motion can be extracted to provide further quantification of data quality (see Results).

### Manual QC review

In addition to the automated QQC and quality measures derived by the preprocessing pipelines, all T1w, T2w, rfMRI and dMRI scans are manually reviewed. In this process trained members of the DPACC MRI team visually inspect 3-plane views using viewers such as FSLeyes and 3D-Slicer, while paging through all frames of the multi-volume rfMRI and dMRI data. For a session with no issues, this manual review takes ~20 min. The output of manual QC is a score for each scan as well as annotations that describe any issues found during the visual inspection. The scores range between 1 and 4, where 4 indicates a high quality scan with no issues, 3 indicates a good quality scan with minor issues (e.g., minor motion, ringing, ghosting or other artifacts) that should nonetheless be usable for most processing pipelines, 2 indicates a lower quality scan with moderate issues that may be unusable in certain processing pipelines, and 1 indicates scans with severe issues that are very likely unusable. For scans rated so far, within each modality fewer than 2% have been rated ‘1’, with 98% rated as either ‘3’ or ‘4’ for the T1w and T2w scans, and greater than 94% rated as either ‘3’ or ‘4’ for the fMRI and dMRI scans.

### Incidental findings

Incidental findings are recorded when the site or DPACC personnel identify a suspected pathology in the obtained images. Each site has their own internal protocol for follow-up of incidental findings, including arranging for a neuroradiologic read if one was not done, and notifying participants when clinically relevant. Additionally, many sites have a routine neuroradiological read for all MRI scans (all sites in PRESCIENT and approximately half of the sites in ProNET). When an incidental finding is identified, the site reports it in a dedicated online form to document follow-up. In addition, if a DPACC MRI team member identifies a suspected pathology during the manual QC process, the relevant site coordinator and principal investigator are notified, and the site follows their internal procedures as if the incidental finding had been identified locally (if not already previously read by a neuroradiologist). The DPACC maintains an aggregated list of incidental findings, along with additional notes, such as the description of the finding, as well as any clinically relevant findings observed in the radiological reads. Finally, the MRI leadership, together with the study leadership, meet periodically to identify if subjects with incidental findings should be excluded from the study since their revealed condition may fall within the exclusion criteria, and to flag cases in which the pathology in question may affect downstream processing of the neuroimaging data (e.g., surface reconstruction or brain parcellation).

## Results

We first briefly describe some of the most common challenges and problems experienced in the MR acquisitions to date. These include incomplete uploads of the imaging data to the network hubs and missing and/or insufficient explanations in the MRI Run Sheet regarding missing, aborted, or repeated scans. To deal with this, a digitized tracker is used in which the DPACC informs the MR Coordinator of each network, who then follows up with the site and ensures resolution of the issue. Scanners that export the DICOM data as one file per slice per volume (e.g., GE) can also pose challenges for certain archiving systems, due to the very large number of files generated per session (We worked with Flywheel to make the Yale XNAT instance for the ProNET hub more robust to handling 100,000+ DICOMs per session, which is the format of the exported data from the GE sites). For this reason, we encourage Siemens XA sites to export their data in ‘Enhanced’ DICOM format (one file per volume, similar in terms of file counts to the “mosaic” format used by Siemens in VE11 for 2D EPI scans). Also, sites on Siemens XA software need to avoid exporting their data immediately after the conclusion of the session, due to a delay of several minutes until all volumes of the final acquisition make it into the on-scanner database. Another common problem is with Siemens sites that run the protocol in a manner that results in changes in the shim between scans, which is detected by the ‘ShimSetting’ check in QQC and remedied by reminding the site personnel of the instructions in the MRI Acquisition SOP. Other, albeit more rare, problems include protocol deviations that are caused either by hardware malfunction or (more frequently) by changes introduced to the protocol by the MR operator at the time of the scan. As soon as these deviations are identified, the site is contacted to either rescan when possible, or to pause scanning and work with the local MR team to resolve the issue.

**Fig. 1:**
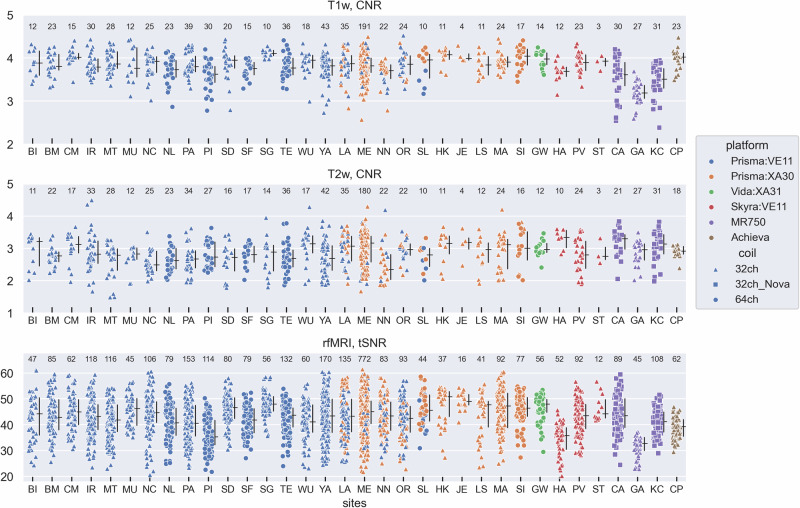
Measures of contrast-to-noise ratio (CNR) for the T1w and T2w scans, and temporal SNR (tSNR) for the rfMRI scans. Values are per scan by site, colored by scanning platform and with different symbols for different types of head coils. The entry for ‘32ch’ coil represents the product 32-channel coil provided by each vendor. Two of the GE MR750 sites (CA, KC) used the Nova Medical 32-channel head coil instead of the GE-provided coil. The ‘64ch’ coil is the Siemens 64-channel “HeadNeck” coil. Measures are “image quality metrics” (IQMs) computed by MRIQC^98^. Numbers at the top of each plot are the scans included in the analysis for each site. If available, both baseline and month 2 visit data were included. For the rfMRI tSNR data, up to 4 scans are included per participant per visit (‘AP’ and ‘PA’ polarity scans, each collected twice per visit). The black vertical lines associated with each site are the inter-quartile range; the horizontal black tick is the median. (Both are without regard to the VE11 vs. XA30 distinction for the Prisma sites that have transitioned software platform).

We explored the cross-site and cross-vendor distribution of various measures generated by MRIQC^98^ for the T1w, T2w, and rfMRI data, and from FSL’s ‘eddy’ tool for the dMRI data (see Methods). Contrast-to-noise ratio (CNR) of the T1w and T2w scans, a measure of the relative separation between gray- and white-matter signal intensity, was reasonably well-harmonized across sites and vendors (Fig. 1). Temporal SNR (tSNR) values in the rfMRI data also have a reasonably consistent distribution across sites, although there are some cross-site differences, such as lower tSNR at sites ‘PI’ (a Siemens Prisma), ‘HA’ (a Siemens Skyra), and ‘GA’ (a GE MR750). The lower tSNR of the rfMRI data from the ‘GA’ site likely reflects the use of the product GE 32-channel head coil, which is known to have a coil configuration that is not as optimal for multi-band imaging of 2D acquisitions with axially-oriented slices. Notably, the two GE MR750 sites using the Nova Medical 32-channel head coil (‘CA’ and ‘KC’) have a tSNR distribution in line with the other sites. The cause for the generally lower tSNR at sites ‘PI’ and ‘HA’ is not known, and may point to a site/scanner specific issue, since the other Prisma and Skyra sites all have a higher median tSNR than those two sites, with no obvious relationship to either head coil (32 vs 64ch) or software version (VE11 vs XA30).

**Fig. 2:**
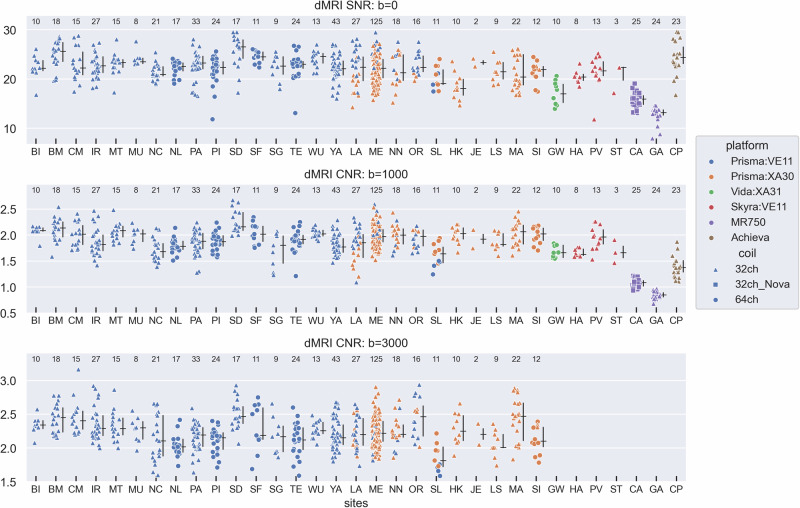
Measures of SNR (*b* = 0) and CNR (*b* = 1000 and 3000 s/mm^2^) for the dMRI acquisitions. Values were computed by FSL's 'eddy' tool. The *b* = 3000 shell was only collected on the Prisma scanner. Similar plots for the other shells (*b* = 200, 500, 2000) are available in Supplemental Fig. S1. No dMRI data is available from site ‘KC’ at this time. See Fig. 1 caption for further details.

Similar scatterplots of SNR (for the *b* = 0 s/mm^2^ shell) and CNR (*b* = 1000 and 3000 s/mm^2^ shells) for the dMRI data are shown in Fig. 2 (with analogous plots for the *b* = 200, 500, and 2000 s/mm^2^ shells in Supplemental Fig. S1). While some site differences are present within a given scanner platform, the big picture from these plots is a gradation across vendors, with CNR in the dMRI data for Siemens platforms higher than the Philips Achieva, which was higher than the GE MR750. This pattern was evident for both the higher (*b* = 1000 and 2000 s/mm^2^) and lower (*b* = 200 and 500 s/mm^2^) shells. Within the GE sites, there is again higher SNR/CNR for the Nova Medical coil relative to the product GE 32-channel head coil (i.e., site ‘CA’ vs ‘GA’). Within the Siemens sites, the SNR/CNR of the non-Prisma (“Tier 2”) platforms (Skyra and Vida) is generally similar to that of the Prisma for the shells in common (*b* ≤ 2000 s/mm^2^), which would not have been possible if we had attempted to collect the *b* = 3000 s/mm^2^ shell on the Tier 2 platforms (due to the substantially longer TE that would have been required).

**Fig. 3:**
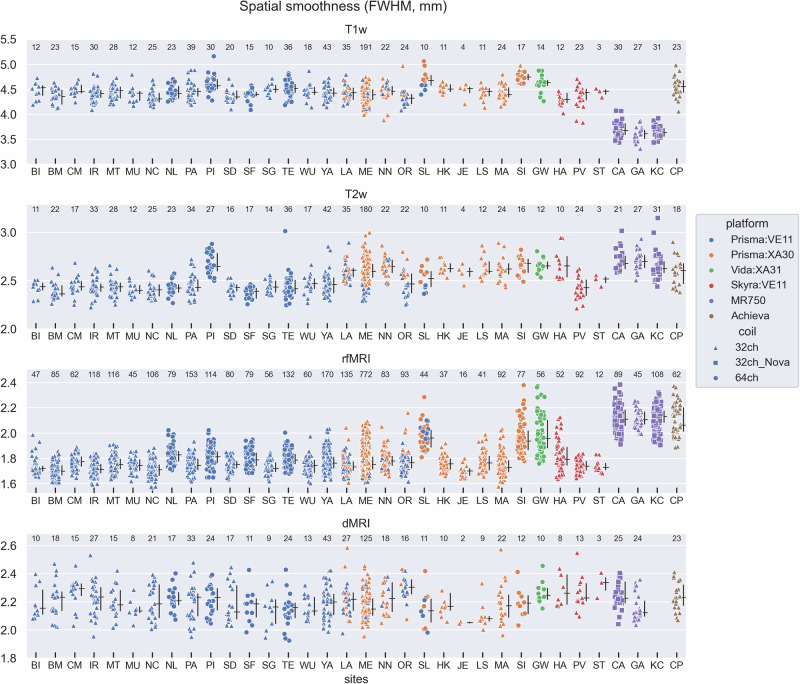
Spatial smoothness estimates (full-width-half-maximum, FWHM) for the data from all 4 MR modalities. The values for the T1w, T2w, and rfMRI scans come from the ‘fwhm_avg’ IQM generated by MRIQC. The values for the dMRI scan were calculated as described in Methods. See Fig. 1 caption for further details.

Another fundamental parameter for understanding possible platform and vendor differences in the MR acquisitions is the empirically estimated spatial smoothness (i.e., full width at half maximum, FWHM) of the data, which is distinct from the requested ‘nominal’ spatial resolution. Importantly, spatial smoothness can easily vary across vendors for a fixed nominal resolution, due to various low-level differences in how the acquisition and reconstruction is implemented by specific vendors. Increases in spatial smoothness correspond to a putative reduction in spatial specificity (which is a “negative”, all else being equal), but may provide some beneficial implicit smoothing (i.e., increased SNR, which is a “positive”, all else being equal). Thus, while empirical smoothness is an important aspect of MRI data that should be quantified, platform and vendor related differences in FWHM should not be treated as indicative of inherently “worse” or “better” performance, which would be overly simplistic. For AMP SCZ, the estimated FWHM of the dMRI data is relatively consistent across platforms and vendors, but the other 3 modalities show differences (Fig. 3). For the T1w acquisitions, the FWHM of the GE MR750 acquisition/reconstruction is clearly lower than the Siemens platforms and the Philips Achieva. Conversely, the FWHM of the rfMRI scans is higher (smoother) for the GE MR750 and Philips Achieva relative to the typical FWHM values from the Siemens platforms, although some Siemens sites clearly have elevated FWHM (e.g., ‘SL’, ‘SI’, and ‘GW’) relative to the others, which appears to be related to the use of the 64-channel Head/Neck coil on the XA platform at those sites (FWHM in the rfMRI data appears moderately elevated on average in Siemens sites using the 64-channel Head/Neck coil on the VE11 platform as well (sites ‘NL’, ‘PI’, ‘SF’, ‘TE’), but less so than the sites using the 64-channel coil on the XA platform (sites ‘SL’, ‘SI’, ‘GW’), suggesting a general effect of coil on FWHM, but a more pronounced coil-by-software interaction within the Siemens sites). For the T2w acquisitions, the data available to date suggest a complicated pattern with (1) elevated FWHM at the GE MR750 and Philips Achieva sites, (2) elevated FWHM at the Siemens sites using the XA software (orange and green in Fig. 3), which seems likely due to a change in the ‘Filter’ setting of the T2w scan necessitated in the Siemens XA protocol (see Supplemental Methods), and (3) elevations in FWHM at sites ‘PI’ (Prisma:VE11 with 64-ch coil) and ‘HA’ (Skyra:VE11 with 32-ch coil), which seem like site-specific variances in the FWHM of the T2w scans unrelated to vendor, software level, or head coil. In summary, FWHM exhibits some complex dependencies on scan modality, vendor and scanning platform, software version, coil, and site that may need to be considered in certain future analyses.

We quantified the percentage of the total variance attributable to the effects of participant, site, and scanner platform by including those as separate random-effect variance components in a linear mixed-effects model (Table 3). A lower variance percentage attributable to sites and scanner platform indicates a reduced contribution of those factors to the overall variability in the QC values. The results in Table 3 are consistent with the scatterplots in Figs. 1–3. For example, FWHM of the T1w and rfMRI data, and SNR/CNR of the *b* = 0 and 1000 shells all have a high percentage (>65%) of their variance related to scanner platform, while CNR/tSNR of the T1w, T2w, and rfMRI data all have participant as their largest variance component (>40%). Residual (i.e., unexplained) variance was the highest variance component for two measures — CNR of the *b* = 3000 shell (40.8%) and FWHM of the dMRI data (62.8%).

**Table 3:** Percentage of variance attributable to Participant, Site, Platform, or Residual, for the measures shown in Figs. 1–3.

	Participant (%)	Site (%)	Platform (%)	Residual (%)
**T1w**				
CNR	**40.4**	7.5	29.3	22.8
FWHM	10.3	3.7	**81.5**	4.6
**T2w**				
CNR	**76.1**	1.5	2.7	19.7
FWHM	**41.5**	14.6	34.2	9.7
**rfMRI**				
tSNR	**48.2**	13.9	3.7	34.2
FWHM	7.2	8.2	**74.4**	10.2
**dMRI**				
SNR, b = 0	9.6	4.3	**66.3**	19.7
CNR, b = 1000	8.1	9.5	**69.1**	13.3
CNR, b = 3000^†^	35.8	23.4	0.0	**40.8**
FWHM	24.2	3.8	9.2	**62.8**

Instances with a given variance percentage greater than 40% are in bold (as an arbitrary threshold to highlight large variance components). ^†^Only two levels to Platform in the b = 3000 shell data (Prisma:VE11 and Prisma:XA30), which only differ in their software version and thus are very similar, such that a random-effect variance of zero was assigned to Platform. Thus, the variance component analysis for the b = 3000 shell should be considered as unique relative to the other shells.

Scatterplots of selected other measures from MRIQC are available for the T1w and rfMRI modalities in Supplemental Figs. S2 and S3, and their associated variance percentages in Table S1. Of note, the estimated intracranial volume fractions for gray- and white-matter in the T1w data are dominated by participant-related variance (63.7 and 71.5%, respectively), with low site- and platform-related variance (<10%), which indicates that the T1w data are reasonably well harmonized at the stage of acquisition on key features related to the actual anatomy (consistent with the T1w CNR results). In the rfMRI data, standardized DVARS, mean framewise displacement, and ghost-to-signal ratio in the phase-encoding axis are all dominated by participant-related variance (>50%), with low site- and platform-related variance (<15%).

## Discussion

We have described various aspects of the MRI component of the broader multi-faceted AMP SCZ protocol for studying individuals at CHR, including (1) the rationale for inclusion of a neuroimaging component in the study, (2) the basic features of the MRI protocol, (3) platform/vendor specific considerations, and (4) the QC processes that are applied upon data ingestion. While it is premature at this time to begin analyzing this rich dataset for project scientific goals, sufficient data have been obtained to begin exploring the impact of site, platform and coil-related effects on available measures. We accomplished this using scatterplots that allow visualization of site, platform, and coil effects, as well as a linear mixed-effects model based variance decomposition into participant, site, and platform variance estimates. This analysis showed that some measures had participant as their largest variance component (which is precisely what we want), such as CNR/tSNR of the T1w, T2w, and rfMRI data, while other measures such as spatial smoothness (FWHM) of the T1w, rfMRI and dMRI data, and SNR/CNR of the dMRI data had either platform or residual (i.e., unexplained) variance as their largest variance components. Variance attributable to site-specific effects was generally small, and never the largest variance component.

Ideally, one wants to minimize site, platform, and residual variance at the acquisition stage while maximizing participant-related variance. Toward this goal, considerable effort went into understanding the different vendors’ platforms and imaging sequences during the creation of the multi-site, multi-vendor MRI protocol for AMP SCZ. However, it is ultimately very challenging currently to minimize platform-related variance in MRI data for certain measures (such as FWHM), due to low-level differences in how different vendors implement the details of the physics of their acquisitions and reconstruction algorithms, or very different hardware capabilities (i.e., differing maximum gradient strength) in the case of CNR/SNR of the dMRI data. The “Pulseq” initiative^102^ to create open-source, vendor-agnostic pulse sequences and reconstructions may be an option in the future to reduce many of these vendor-specific differences^103^ and provide a mechanism for truly harmonized acquisitions across platforms and vendors. Meanwhile, it would be interesting and informative to compare our quantitative results to other large-scale, multi-site, multi-vendor imaging protocols, but are not aware of a similar variance quantification by other such studies at this time.

A key component of the AMP SCZ initiative is its large prospective sample size (~2000 CHR individuals), which will be important for the generalizability and robustness of its imaging findings^81^. Notably, the size of this CHR sample will be substantially larger than the existing largest CHR studies such as NAPLS-2 and NAPLS-3^104^, PACE^105^, PRONIA^106^, PSYSCAN^107^, and SHARP^108^, which range between about 250–700 CHR individuals. Further, since all CHR participants in AMP SCZ are planned to receive all assessments, analyses across the various domains (e.g., clinical, cognitive, neuroimaging) will be possible with an equally large sample size. To date, other similarly large CHR samples have only been aggregated via retrospective pooling of multiple, smaller independent studies, such as the effort by Malda et al.^109^, which did not include imaging data, and the Enhancing Neuroimaging Genetics Through Meta-analysis (ENIGMA) Clinical High Risk for Psychosis Working Group^12,110–112^, which to date has only analyzed morphometric measures derived from FreeSurfer (cortical thickness, surface area, and subcortical volume). These ENIGMA studies (with CHR samples of ~1200-1800 individuals), while finding statistically significant differences between CHR and controls, report only small differences (decreases) in cortical thickness in CHR individuals relative to controls in even the most-different regions (Cohen’s d of 0.22 or less)^12,111^. This finding of small effect sizes in ENIGMA is consistent with the general observation that the reported effect sizes and classification accuracy of potential imaging biomarkers have often decreased with increasing sample size^81,113^. While it remains possible that other MR modalities (e.g., fMRI and dMRI) may exhibit larger regional effects, these ENIGMA studies demonstrate the likely importance of multivariate, higher-dimensional analyses involving multiple MR modalities to discover subtle patterns of the risk of conversion to psychosis. Additionally, these ENIGMA analyses involved only cross-sectional data. The longitudinal neuroimaging component in AMP SCZ may capture subtle differences in the trajectories between individuals^39^ and thereby facilitate better discrimination.

An additional advantage of the large size of the AMP SCZ dataset is that it will be possible to consider various modeling approaches for dealing with the remaining site- and platform-related variance, including (1) simple inclusion of fixed-effect confounds within ordinary least square regression^114^, (2) harmonization using ComBat – an empirical Bayes framework that adjusts the location (mean) and scale (variance) of the data^115–119^, possibly extended with a generalized additive model that allows for nonlinear age effects^120^, (3) spherical harmonic-based retrospective harmonization of dMRI data^121–123^, or (4) deep learning approaches^124–127^.

There are several limitations of the neuroimaging protocol for AMP SCZ. The 1 h time limit for the duration of scanning session, along with the challenges of implementing a protocol across multiple vendors, precluded us from including additional MR modalities, such as an arterial spin labeling (ASL) scan to measure cerebral blood flow and MR spectroscopy (MRS) to measure certain neurochemicals believed to be related to neuronal and axonal health. While MRS, and especially ASL, studies in CHR are less common^128,129^, their inclusion might have yielded valuable additional information regarding brain correlates of the CHR state or psychosis conversion. Similarly, time limitations restricted the resting-state fMRI data to just 20 min in total per visit, which is less than the 30 min that has been suggested to increase the reliability (and thus predictive power) of connectivity estimated from rfMRI data^130^. However, recent modeling of the tradeoffs between sample size and rfMRI scan time per participant suggests that for a large N study, 20 min may be near optimal, and certainly not dramatically worse than 30 min, in terms of the ability of rfMRI connectivity to predict cognitive and mental health phenotypes^131^. For the dMRI scans, a “two tier” protocol was necessary in order to take advantage of the capabilities of the Siemens Prisma scanner, but which will nonetheless complicate the analysis of the dMRI data and reduce the sample size for dMRI analyses that wish to take advantage of the *b* = 3000 s/mm^2^ shell facilitated by the Prisma scanner. A limitation of the variance component analyses in this manuscript is their preliminary nature. In particular, at the time of analysis a small number of sites that were onboarded later into the study had not yet contributed any participants, or had only collected a small number of participants. We plan to refresh these analyses and extend them to additional variables as the study progresses. Also, different types of preprocessing and denoising would alter the variance ratios, although the main conclusions should remain broadly similar.

The potential of various medications to alter or normalize MRI findings is a complicated issue that will pose challenges for the interpretation of the MRI results^132–134^. Prescribed antipsychotic, antidepressant, and other psychotropic medications, including dosage and adherence, are documented for lifetime use at study entry and at each visit over follow-up^26^. CHR individuals with current or more than minimal past antipsychotic use at study entry are excluded from the study, as are community controls with current treatment with any psychotropic medication^29^. We will attempt to account for differences in medication across individuals by converting to both current and lifetime dose equivalents (e.g.^135^) and then using those equivalents as covariates of no interest or to explore the impact of different medication classes on the MRI results. Additionally, the large size of AMP SCZ may result in sufficient sub-samples to directly compare the possible impact of certain medications against no medication and each other, at least for the more common types of medications.

By describing the process and reasoning by which the AMP SCZ neuroimaging protocol was created, we hope that this paper will be valuable for other studies that plan complex multi-site, multi-vendor imaging studies in clinical populations. Additionally, the description and initial quantification of this protocol should be useful for researchers who will be working directly with its multi-modal MR data, which will be publicly available. Some studies may want to consider outright adoption of the AMP SCZ imaging protocol, which may be especially valuable for studies that would benefit from aggregation of larger datasets and joint analyses with the AMP SCZ data, including the development of normative models of brain morphometry or function in individuals with CHR, comparison with first episode or established psychosis, training machine learning algorithms and advanced statistical models to identify relationships between brain data and clinical outcomes, or just small imaging studies that would benefit from direct comparison to the AMP SCZ data. Researchers using the AMP SCZ neuroimaging protocol are encouraged to collect a sufficient number of control individuals in their studies to check for differences relative to the AMP SCZ controls, and to flexibly support certain harmonization approaches. Studies that use the AMP SCZ protocol can also benefit from the additional available resources such as SOPs, importable protocols, and processing code that are available at the AMP SCZ website (www.ampscz.org). Overall, the AMP SCZ consortium is an exciting step forward in CHR research, and we are optimistic that this MRI protocol, both as a separate data modality, and in combination with other data domains collected in AMP SCZ, will play an important role in understanding the neurobiology of CHR and factors that may predict clinical and treatment outcomes.

## Methods

All scans analyzed in this paper were reconstructed on the scanner using the vendor’s on-scanner implementation of receive-coil bias field correction (i.e., PreScan Normalize for Siemens, PURE for GE, CLEAR for Philips). The T1w, T2w, and rfMRI data were processed through MRIQC (v. 22.0.6)^98^ using its default settings. Our primary measures of interest were contrast-to noise ratio (CNR) between gray-matter and white-matter for the T1w and T2w scans, temporal signal-to-noise (tSNR) for the rfMRI scans, and spatial smoothness (full-width-half-maximum, FWHM) for all modalities, although some additional measures are included in the Supplemental Results. For the T2w scans, we found that the FSL FAST tissue segmentation used by MRIQC typically resulted in a segmentation volume whose numeric labels did not agree with the assumptions coded into MRIQC, resulting in invalid calculations and variable labels for any MRIQC measure derived from the tissue classification. We corrected the CNR calculation for the T2w data using an appropriate remapping of the tissue classes. For the dMRI data, SNR (for the *b* = 0 shell) and CNR (for the *b* = 200-3000 shells) was obtained from FSL’s ‘eddy’ tool^136^, with FWHM estimated using AFNI’s ‘3dFWHMx’ tool. See Supplemental Methods for further details regarding both the T2w and dMRI processing.

To derive the dataset used for analysis, we applied the data selection and cleaning procedures outlined in the Supplemental Methods (see *Data Selection and Cleaning)* to the data provided by the DPACC for sessions acquired prior to December 2023. The final dataset used for analysis contained 891 T1w scans, 856 T2w scans, 3408 rfMRI runs, and 663 dMRI scans, representing 601, 591, 607, and 497 unique individuals in each modality, and 290, 265, 287, and 166 individuals with both baseline and 2-month visit data in each modality, respectively.

We used a linear mixed-effects (LME) model to estimate the variance attributable to the effects of participant, site, and scanner platform. There were 34 sites with available data, and 6 modeled scanner platforms, with the VE11 and XA30 software versions of the Siemens Prisma modeled as two different platforms and the Siemens Vida (XA31), Siemens Skyra (VE11), GE MR750 (all on a variant of DV26), and Philips Achieva (5.7.1) as the other 4 platforms. The LME model was implemented within the Pymer4 package^137^, which provides an interface between Python and the ‘lmer’ function within the ‘lme4’ package of R. The model included ‘sex’ and ‘age at scan’ as fixed effects, to remove variance potentially related to basic demographic differences in the recruitment profile across sites. Specifically, the model formula was:

measure ~ sex + age_at_scan + (1|participant) + (1|site) + (1|platform)

We computed the total variance as the sum of the four variance components (including the residual, i.e., “unexplained”, variance) and report each component as a percentage of the total variance. For the T1w, T2w, and dMRI data, estimating participant-related variance was possible due to the inclusion of both baseline and 2-month visit data from many of the participants (see N’s above). For the rfMRI data, there were up to 4 rfMRI runs per session, with two pairs of runs per session, each with an ‘AP’ and ‘PA’ phase-encoding polarity (see Table 2). Inclusion of scan polarity (‘AP’ or ‘PA’) or visit (baseline or 2-month) as additional fixed-effect factors in the model for the rfMRI data had very little impact on the variance percentages.

## Supplementary information

Supplemental Protocol Details Spreadsheet

Supplemental Material

AMP SCZ Member List & Affiliations

## Data Availability

In alignment with the AMP SCZ commitment to open science, and ensuring transparency, reproducibility, and accessibility, the large amount of data being collected for AMP SCZ will be publicly released through the National Institute of Mental Health Data Archive (NDA) AMP SCZ Data Repository. This will include unprocessed image data (that has been QC’ed and curated) in the form of BIDS organizational structure^97^. Data will be released in discrete batches. For example, “Release 2.0” is already available, which includes anonymized BIDS data with QC scores of 3 or 4, with baseline imaging data from 425 individuals, and imaging data from the 2-month visit for 196 individuals. Future releases will include additional participants, as well as additional processing steps and derived measures that will be calculated for the scientific aims of AMP SCZ, QC information, and additional metadata. Interested researchers can request access to the data using the standard data request page available at nda.nih.gov/ampscz. Each participant provided oral and written informed consent. The project was approved by the governing institutional review board at each site and is registered at clinicaltrials.gov (NCT05905003).
