# Supplementary material for: The MR neuroimaging protocol for the Accelerating Medicines Partnership® Schizophrenia Program
Source: Schizophrenia (Heidelb). 2025 Apr 2;11(1):52. doi: 10.1038/s41537-025-00581-6 (PMC11965426; doi:10.1038/s41537-025-00581-6)
Supplement: Supplementary file 2 — Supplemental Material [file 41537_2025_581_MOESM2_ESM.docx]

**Supplemental Information for**

**The MR Neuroimaging Protocol for the Accelerating Medicines Partnership**® **Schizophrenia Program**

Michael P. Harms, Kang-Ik K. Cho, Alan Anticevic, Nicolas R. Bolo, Sylvain Bouix, Dylan Campbell, Tyrone D. Cannon, Guillermo Cecchi, Mathias Goncalves, Anastasia Haidar, Dylan E. Hughes, Igor Izyurov, Omar John, Tina Kapur, Nicholas Kim, Elana Kotler, Marek Kubicki, Joshua M. Kuperman, Kristen Laulette, Ulrich Lindberg, Christopher Markiewicz, Lipeng Ning, Russell A. Poldrack, Yogesh Rathi, Paul A. Romo III, Zailyn Tamayo, Cassandra Wannan, Alana Wickham, Walid Yassin, Juan Helen Zhou, Jean Addington, Luis Alameda, Celso Arango, Nicholas J.K. Breitborde, Matthew R. Broome, Kristin S. Cadenhead, Monica E. Calkins, Eric Yu Hai Chen, Jimmy Choi, Philippe Conus, Cheryl M. Corcoran, Barbara A. Cornblatt, Covadonga M. Diaz-Caneja, Lauren M. Ellman, Paolo Fusar-Poli, Pablo A. Gaspar, Carla Gerber, Louise Birkedal Glenthøj, Leslie E. Horton, Christy Lai Ming Hui, Joseph Kambeitz, Lana Kambeitz-Ilankovic, Matcheri S. Keshavan, Sung-Wan Kim, Nikolaos Koutsouleris, Jun Soo Kwon, Kerstin Langbein, Daniel Mamah, Daniel H. Mathalon, Vijay A. Mittal, Merete Nordentoft, Godfrey D. Pearlson, Jesus Perez, Diana O. Perkins,  Albert R. Powers III, Jack Rogers, Fred W. Sabb, Jason Schiffman, Jai L. Shah, Steven M. Silverstein, Stefan Smesny, William S. Stone, Gregory P. Strauss, Judy L. Thompson, Rachel Upthegrove, Swapna Verma, Jijun Wang, Daniel H. Wolf, Rene S. Kahn, John M. Kane, Patrick D. McGorry, Barnaby Nelson, Scott W. Woods,  Martha E. Shenton, Stephen J. Wood, Carrie E. Bearden, Accelerating Medicines Partnership® Schizophrenia (AMP® SCZ), Ofer Pasternak

**Contents**

Supplemental Results:

Figure S1: Scatterplots of CNR for the b=200, 500, and 2000 s/mm2 shells for the dMRI acquisitions.

Figure S2: Scatterplots of selected other measures from MRIQC for the T1w scans.

Figure S3: Scatterplots of selected other measures from MRIQC for the rfMRI scans.

Table S1: Percentage of variance attributable to Participant, Site, Platform, or Residual, for the measures shown in Figures S1-S3.

Supplemental Methods:

Issues with segmentation of T2w scan

dMRI scan processing

Data Selection and Cleaning

**SUPPLEMENTAL RESULTS


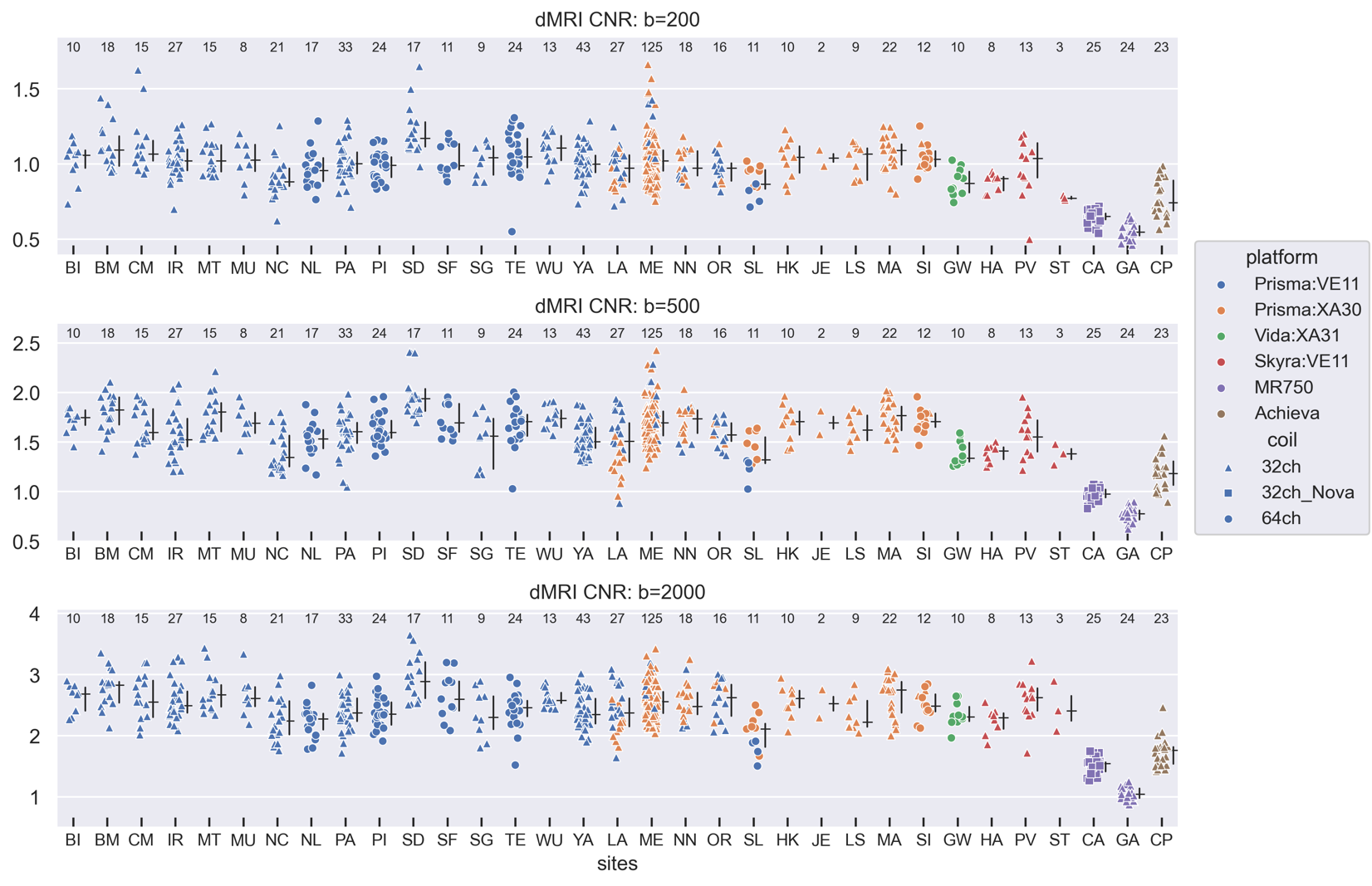
Figure S1:** Scatterplots of CNR for the b=200, 500, and 2000 s/mm^2^ shells for the dMRI acquisitions, as computed by FSL’s ‘eddy’ tool. Similar plots for the other shells are available in Figure 2. See Figure 1 caption for additional details.

**
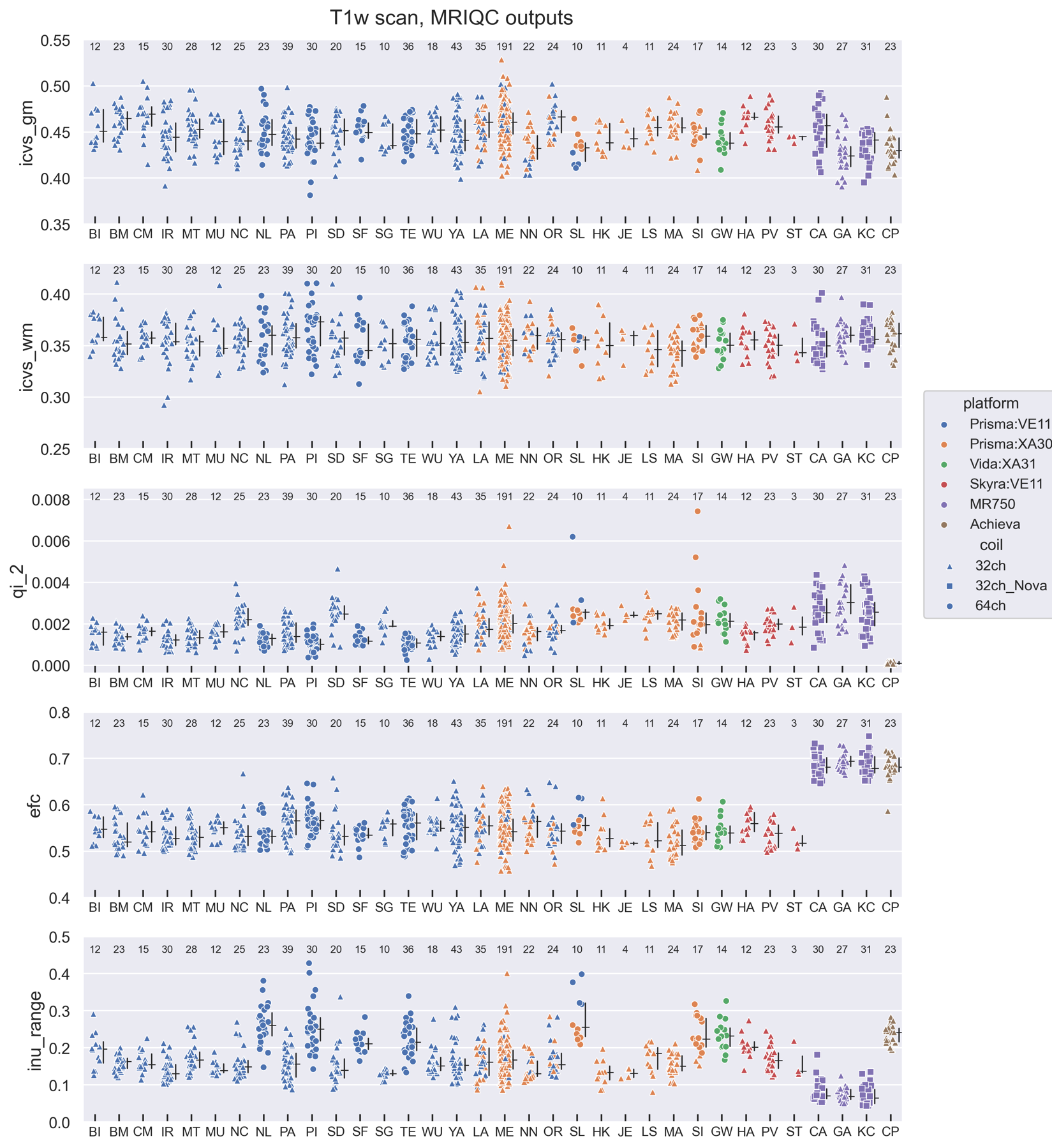
Figure S2:** Scatterplots of selected other measures from MRIQC^1^ for the T1w scans. These 5 measures plus ‘cnr’ and ‘fwhm’ (Figures 1 and 3) had an absolute Spearman correlation < 0.5 with each other, indicating that those 7 measures are capturing relatively distinct features of the T1w scans. icvs_gm and icvs_wm: intracranial volume fraction of gray-matter and white-matter, respectively; qi_2: Mortamet’s quality index 2 – a measure of the goodness of fit of a χ^2^ distribution on a background (air) mask^2^; efc: entropy focus criterion – a normalized version of the Shannon entropy of the voxel intensities; inu_range: the difference between the 95^th^ and 5^th^ percentiles of the estimated bias field (intensity non-uniformity). See Figure 1 caption for additional details.


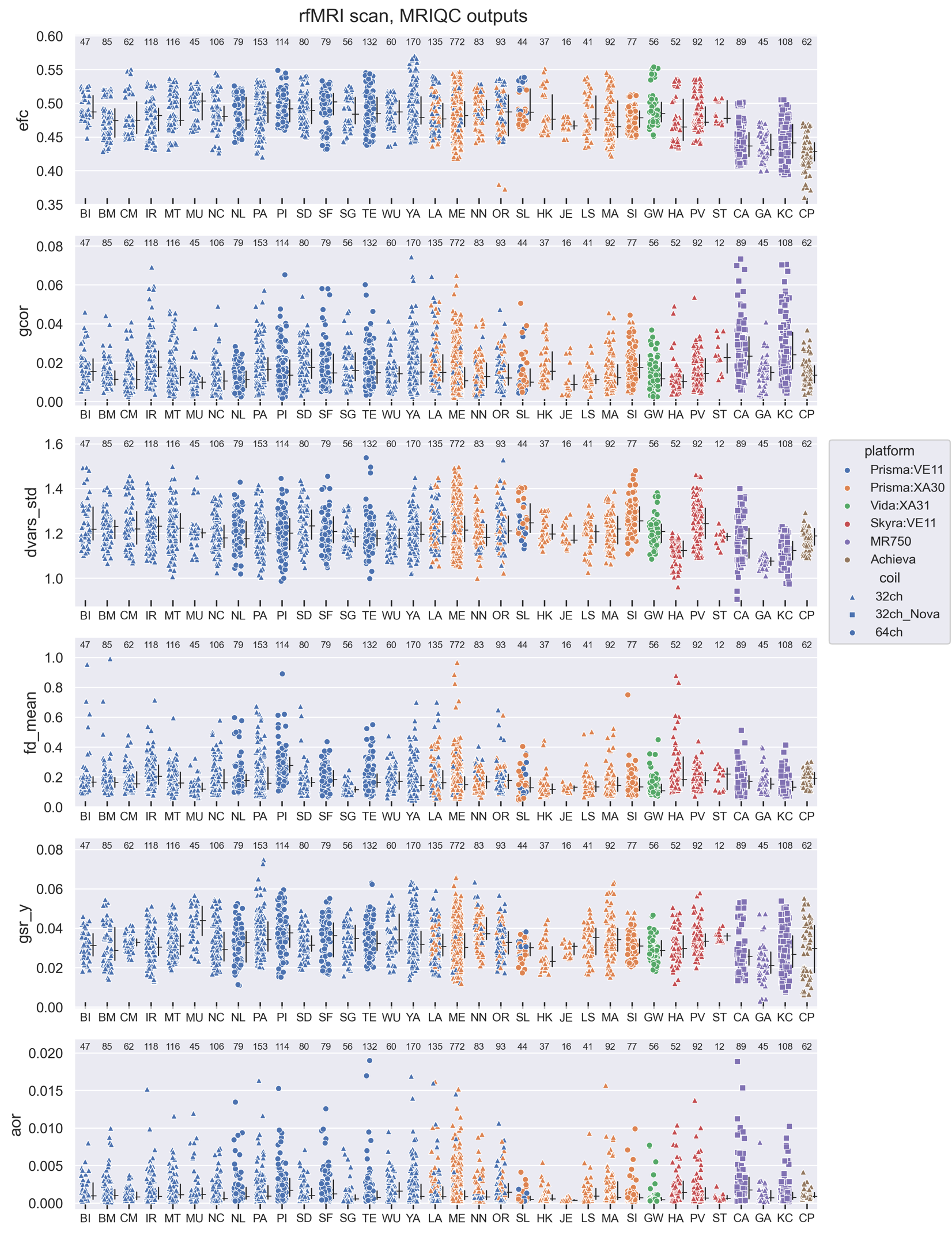


**Figure S3:** Scatterplots of selected other measures from MRIQC^1^ for the rfMRI scans. These 6 measures plus ‘tsnr’ and ‘fwhm’ (Figures 1 and 3) had an absolute Spearmean correlation < 0.7 with each other, indicating that those 8 measures are capturing relatively distinct features of the rfMRI scans. efc: entropy focus criterion – a normalized version of the Shannon entropy of the voxel intensities; gcor: global correlation, computed using AFNI’s ‘@compute_gcor’; dvars_std: a version of DVARS^3^ normalized by the standard deviation of the temporal difference time series; fd_mean: mean of framewise displacement^3^ across the time series; gsr_y: ghost-to-signal ratio along the phase-encoding axis (AP/PA); aor: mean fraction of outliers per fMRI volume from AFNI’s ‘3dToutcount’. See Figure 1 caption for additional details.

|  | Participant (%) | Site (%) | Platform (%) | Residual (%) |
| --- | --- | --- | --- | --- |
| **T1w** |  |  |  |  |
| icvs_gm | **63.7** | 4.2 | 8.6 | 23.6 |
| icvs_wm | **71.5** | 0.8 | 0.0 | 27.7 |
| qi_2 | 20.7 | 13.3 | **47.5** | 18.6 |
| efc | 12.0 | 0.6 | **86.3** | 1.12 |
| inu_range | 20.1 | 22.7 | **48.9** | 8.3 |
| **rfMRI** |  |  |  |  |
| efc | 26.6 | 0.8 | **69.0** | 3.6 |
| gcor | 36.9 | 6.8 | 6.5 | **49.8** |
| dvars_std | **52.2** | 4.3 | 15.0 | 28.5 |
| fd_mean | **57.1** | 5.5 | 0.0 | 37.4 |
| gsr_y | **58.7** | 5.3 | 13.5 | 22.6 |
| aor | 30.4 | 1.5 | 0.0 | **68.1** |
| **dMRI** |  |  |  |  |
| CNR, b=200 | 8.8 | 7.6 | **55.7** | 27.9 |
| CNR, b=500 | 9.4 | 11.6 | **62.4** | 16.6 |
| CNR, b=2000 | 10.4 | 8.2 | **67.9** | 13.5 |

**Table** **S1**: Percentage of variance attributable to Participant, Site, Platform, or Residual, for the measures shown in Figures S1-S3. Instances with a given variance percentage greater than 40% are in bold. Note that qi_2, efc, and inu_range are all expected to be sensitive to the specific details and effectiveness of each vendor’s on-scanner implementation of receive-coil bias-field correction (i.e., PreScan Normalize for Siemens, PURE for GE, CLEAR for Philips). Thus, it is not surprising that those measures show large percentages of variance attributable to platform effects.

**SUPPLEMENTAL METHODS**

*Issues with segmentation of T2w scan*For the T2w scans, we found that the FSL FAST tissue segmentation used by MRIQC typically resulted in a segmentation volume whose numeric labels did not agree with the assumptions coded into MRIQC. Specifically, MRIQC assumes a mapping between FAST segmentation labels and tissue classification that is appropriate for T1w data: 0 = background, 1 = CSF, 2 = GM (gray-matter), 3 = WM (white matter). However, in our T2w data, we found that the *typical* mapping returned by FAST was 0 = background, 1 = GM, 2 = WM, 3 = CSF. Consequently, the labels used by MRIQC for the T2w tissue classes were not anatomically correct (the ‘csf’ label within MRIQC was typically actually gray-matter, the ‘gm’ label within MRIQC was typically white-matter, and the ‘wm’ label within MRIQC was typically CSF). This resulted in invalid default calculations and labels for any measure based on the tissue classification of the T2w scan. We corrected the CNR calculation for the T2w data using the same CNR formula internal to MRIQC after an appropriate remapping of the tissue classes. However, further complicating the situation, we found that the FAST segmentation was somewhat unreliable on our T2w data, with some portions of WM sometimes labeled as class 3 (so that some anatomical WM was correctly labeled as ‘wm’). In a small number of cases, the labels for CSF and WM even swapped entirely (so that CSF was labeled as ‘gm’). We excluded these cases in an automatic fashion through the criteria stated below (in *Data Selection and Cleaning*). Nonetheless, we limited the presented measures for the T2w data to our (corrected) CNR measure, and FWHM (which is independent of the tissue classification).

*dMRI scan processing*For processing the dMRI data, Gibb’s ringing artifact was first removed using ‘unring’^^[[1]](#footnote-1)^^. The concatenated dMRI scans (both “b=0” scans and main dMRI scan) were then processed with ‘eddy’^4^ in FSL 6.0.6.5, with the features of outlier replacement^5^, slice-to-volume motion modeling^6^, and susceptibility-by-movement correction^7^. The specific command was:
eddy_cuda10.2 \

    --imain={input_img} \

    --bvecs={bvec} \

    --bvals={bval} \

    --mask={mask} \

    --acqp={acqp} \

    --index={index} \

    --estimate_move_by_susceptibility \

    --niter=6 \

    --fwhm=10,6,2,0,0,0 \

    --residuals \

    --out={out} \

    --ol_nstd=5 \

    --repol \

    --topup={topup} \

    --data_is_shelled \

    --slspec={slspec} \

    --ol_type=both \

    --mporder=9 \

    --cnr_maps
where {input_img} was the concatenated dMRI scans. The output of ‘eddy’ includes an ‘eddy_cnr_maps’ volume in which the first volume contains a map of the SNR of the b=0 volumes (defined as mean of the b=0 volumes divided by standard deviation of the b=0 volumes) and the other volumes contain CNR maps of each of the diffusion-weighted shells [defined as the standard deviation of the Gaussian Process (GP) predictions within ‘eddy’ divided by the standard deviation of the residuals (i.e., the difference between the observations and GP predictions)]^[[2]](#footnote-2)^. From the SNR/CNR maps we then computed the mean value for each shell within the dMRI brain mask. The use of outlier replacement (--repol flag) combined with slice-to-volume motion modeling (--mporder flag) resulted in some dMRI scans being unable to complete ‘eddy’ processing, due to an “Unable to find volume with no outliers in shell” error. This is due to the small number of volumes collected in the low b-value shells (b=200 and 500) combined with the specifics of the outlier identification algorithm. The developer of ‘eddy’ is aware of this issue and is working on a solution so that we will be able to process those scans without excluding the b=200 and 500 shells.

For estimating FWHM of the dMRI data, we used the following command from AFNI:
3dFWHMx -input {eddy_residual_map} -mask {eddy_mask} -ACF NULL -ShowMeClassicFWHM
where {eddy_residual_map} is the ‘eddy_residuals’ volume output by the inclusion of the “--residuals" flag. In this manuscript we use the result of the “Classic” FWHM estimate from 3dFWHMx (computed from nearest-neighbor differences), rather than the (newer) autocorrelation function (ACF) approach, since the “Classic” estimate is what is implemented inside of MRIQC. The FWHM values from the ACF approach are larger, but highly correlated with those from the “Classic” approach (r = 0.99 in the dMRI data). All reported FWHM values are the geometric average across the 3 spatial axes.

*Data Selection and Cleaning*Starting with sessions acquired prior to December 2023 we: (1) selected the on-scanner bias-field corrected reconstruction for the T1w and T2w data, (2) removed instances of more than two T1w, T2w, or dMRI runs per session, or more than four rfMRI runs per session (due to uncertainty regarding which should be favored at this time), (3) removed scans that had ratings of ‘1’ or ‘2’ following manual review (< 4% of each modality), but preserving for analysis those scans that the DPACC had not yet had a chance to manually review (~23% of each modality), (4) eliminated any scans with incorrect acquisition parameters (1 T1w scan; 2 T2w scans; and 12 rfMRI runs spanning 4 sessions), (5) required at least 300 frames collected for the rfMRI runs (eliminated 4 runs), and finally (6) removed a small number of scans that were clear visual outliers across the sites as a whole. Specifically, we applied the following conditions sequentially to eliminate outliers (see <https://mriqc.readthedocs.io> for descriptions of these additional metrics): (i) for the T1w scans, ‘inu_range’ > 0.5 (2 scans); (ii) for the rfMRI scans, ‘fd_mean’ > 1.0, ‘gsr_y’ > 0.08, ‘aor’ > 0.02, ‘aqi’ > 0.08, ‘gcor’ > 0.08, and ‘dvars_vstd’ > 1.2, which eliminated 5, 4, 10, 2, 4, and 1 rfMRI run(s), respectively; (iii) for the dMRI scans, ‘abs_motion’ > 3.0 (30 scans) and ‘rel_motion’ > 1.0 (6 scans), estimates of “absolute” and “relative” motion provided by ‘eddy’; additionally we eliminated all dMRI scans from site ‘KC’ due to application of the wrong diffusion-vector file and 19 scans from site ‘ME’ due to issues related to a cracked gradient coil and its subsequent replacement; (iv) for the T2w scans, we were slightly more permissive in excluding scans due to the aforementioned issues with its tissue segmentation, and used the following exclusions: ‘icvs_csf’ > 0.3, ‘icvs_gm’ < 0.2, ‘icvs_wm’ < 0.3, ‘cjv’ > 10, ‘inu_range’ > 0.5, ‘cnr’ (corrected) < 1.0, which eliminated 27, 6, 3, 10, 7, and 1 scan(s), respectively. For the intracranial volume fraction (icvs) criteria listed here for the T2w data, for interpretational convenience we have changed the labels (csf, gm, wm) to reflect what would be the correct anatomy per the *typical* classification returned by FSL FAST.

**References**

1 Esteban, O. *et al.* MRIQC: Advancing the automatic prediction of image quality in MRI from unseen sites. *PLoS One* **12**, e0184661 (2017). <https://doi.org/10.1371/journal.pone.0184661>

2 Mortamet, B. *et al.* Automatic quality assessment in structural brain magnetic resonance imaging. *Magn Reson Med* **62**, 365-372 (2009). <https://doi.org/10.1002/mrm.21992>

3 Power, J. D., Barnes, K. A., Snyder, A. Z., Schlaggar, B. L. & Petersen, S. E. Spurious but systematic correlations in functional connectivity MRI networks arise from subject motion. *Neuroimage* **59**, 2142-2154 (2012).

4 Andersson, J. L. R. & Sotiropoulos, S. N. An integrated approach to correction for off-resonance effects and subject movement in diffusion MR imaging. *Neuroimage* **125**, 1063-1078 (2016). <https://doi.org/10.1016/j.neuroimage.2015.10.019>

5 Andersson, J. L. R., Graham, M. S., Zsoldos, E. & Sotiropoulos, S. N. Incorporating outlier detection and replacement into a non-parametric framework for movement and distortion correction of diffusion MR images. *Neuroimage* **141**, 556-572 (2016). <https://doi.org/10.1016/j.neuroimage.2016.06.058>

6 Andersson, J. L. *et al.* Towards a comprehensive framework for movement and distortion correction of diffusion MR images: Within volume movement. *Neuroimage* **152**, 450-466 (2017). <https://doi.org/10.1016/j.neuroimage.2017.02.085>

7 Andersson, J. L. R., Graham, M. S., Drobnjak, I., Zhang, H. & Campbell, J. Susceptibility-induced distortion that varies due to motion: Correction in diffusion MR without acquiring additional data. *Neuroimage* **171**, 277-295 (2018). <https://doi.org/10.1016/j.neuroimage.2017.12.040>

1. <https://bitbucket.org/reisert/unring>; <https://doi.org/10.48550/arXiv.1501.07758> [↑](#footnote-ref-1)
2. The release notes for FSL mention a “bug fix” to ‘eddy’ in FSL 6.0.7.8 (<https://fsl.fmrib.ox.ac.uk/fsl/docs/#/development/history/changelog-6.0.7.8>) that restored the CNR calculation in ‘eddy’ to its intended form of **std**(signal)/**std**(residuals). We empirically confirmed that the 6.0.6.5 version that we used to generate our CNR results also used the **std**(signal)/**std**(residuals) form, and not the **range**(signal)/**std**(residuals) form, which is the “bug” that found its way into some of the ‘eddy’ versions prior to 6.0.7.8 (but sometime after version 6.0.6.5). [↑](#footnote-ref-2)
